# A highly potent and selective inhibitor Roxyl-WL targeting IDO1 promotes immune response against melanoma

**DOI:** 10.1080/14756366.2018.1471688

**Published:** 2018-06-22

**Authors:** Guangwei Xu, Tianqi Wang, Yongtao Li, Zhi Huang, Xin Wang, Jianyu Zheng, Shengyong Yang, Yan Fan, Rong Xiang

**Affiliations:** a Department of Medicinal Chemistry, School of Medicine, Nankai University, Tianjin, China;; b State Key Laboratory and Institute of Elemento-Organic Chemistry, Collaborative Innovation Center of Chemical Science and Engineering, Nankai University, Tianjin, China;; c State Key Laboratory of Biotherapy and Cancer Center, West China Hospital, Sichuan University and Collaborative Innovation Center for Biotherapy, Chengdu, China;; d State Key Laboratory of Medicinal Chemical Biology, Tianjin, China;; e 2011 Project Collaborative Innovation Center for Biotherapy of Ministry of Education,Tianjin, China

**Keywords:** IDO1, inhibitor, melanoma, immunotherapy

## Abstract

Indoleamine 2,3-dioxygenase 1 (IDO1) activity links to immune escape of cancers. Inhibition of IDO1 provides a new approach for cancer treatment. Most clinical IDO1 drugs show marginal efficacy as single agents. On basis of molecular docking and pharmacophore modelling, a novel inhibitor Roxyl-WL was discovered with a half maximal inhibitory concentration (IC50) value of 1 nM against IDO1 and 10–100-fold increased potent activity compared with IDO1 drugs in clinical trials. Roxyl-WL displayed excellent kinase spectrum selectivity with no activity out of the 337 protein kinases. *In vitro*, Roxyl-WL effectively augmented the proliferation of T cells and reduced the number of regulatory T cell (Tregs).When administered to melanoma (B16F10) tumor-bearing mice orally, Roxyl-WL significantly suppressed tumor growth and induced immune response.

## 1. Introduction

As a hallmark of cancer, tumor immune escape brings many difficulties and troubles for cancer therapy. Indoleamine 2,3-dioxygenase 1 (IDO1) has been proved a monomeric heme-containing oxidoreductase that catalyzes tryptophan (Trp) degradation in the first and rate-limiting step along the kynurenine (Kyn) pathway^1^. The catabolism of Trp is an important mechanism in immune tolerance^2–4^. By depleting Trp, IDO1 blocks the proliferation of T lymphocytes, enhances the immunosuppression mediated by regulatory T cells (Tregs) and suppresses the immune response, which plays an essential role in tumor escape^5^^,^^6^. IDO1 overexpresses in many types of human malignancies^7–11^, such as melanoma^12^. The overexpression of IDO1 in tumor cells, as well as in the dendritic cells (DCs) that localise to the tumor draining lymph nodes, has been shown to be correlated with reduced overall survival in patients^7^. As previously reported, inhibition of IDO1 produces significant anticancer effects. IDO1 linking the process of immune evasion by tumors represents a novel target for cancer therapy.

To the best of our knowledge, only several IDO1 inhibitors have entered clinical trials, such as INCB024360 (Epacadostat)^13–15^, the imidazole NLG919^16^^,^^17^ and NLG-8189 (Indoximod, 1-Methyl-tryptophan (1-MT); Supplementary Figure S1). The most widely studied of these was 1-MT. It has been reported that 1-MT is bioactive and selective but is a rather low potency compound. One of the most advanced INCB024360 is an orally inhibitor of the IDO1 protein with an half maximal inhibitory concentration (IC50) value of 12 nM^18^. In addition, despite its good potency, the objective responses were not observed in clinical trials with single-agent, which result a relatively high dose to reach full inhibition of the target and more side effects. NLG919 developed by NewLink Genetics is undergoing phase I clinical trials in the treatment of recurrent advanced solid tumors. NLG919 showed inhibition of IDO1 in enzymatic assay with half maximal effective concentration (EC_50_) value of 75 nM and only a few efficacy reports of NLG919 were obtained at AACR meeting^19^. To date, there is no inhibitors listed approved by the Food and Drug Administration. Therefore, it is necessary and imminent to develop new IDO1 inhibitors.

Herein, we report our efforts in the virtual screen, synthesis, and biological evaluation of a highly potent and selective IDO1 inhibitor Roxyl-WL with a novel scaffold structure. Molecular docking and pharmacophore modeling screening were applied at the beginning to choose potential lead compounds and improve the efficiency of lead discovery.

## 2. Materials and methods

### 2.1. Molecular docking

All the calculations were carried out using the platform of Discovery Studio 3.1 (DS 3.1, Accelrys Inc., San Diego, CA). The receptor protein was prepared by the DS 3.1 software package with standard preparation procedures. Compound libraries used for docking were prepared with the “Prepare Ligands” module in DS 3.1.

### 2.2. Pharmacophore modeling

Pharmacophore-model-based virtual screening was performed by using the complex structure of IDO1 and its ligands. The pharmacophore features were created manually with the “Query Featurs” module in DS 3.1. The 3 D database was built from the top ranking compounds, which was screened by molecular docking.

### 2.3. Treg cell experiment

Lymphocytes of mice were prepared from splenocytes of a common inbred strain of laboratory mouse C57BL/6, resuspended in Roswell Park Memorial Institute medium (RPMI) 1640 medium supplemented with 10% fetal bovine serum, 1% L-glutamate, 1% penicillin and streptomycin, 100 U/mL interleukin-2, 2 × 10^6^/well in six-well plate with 1-MT 500 μM or Roxyl-WL 500 nM. After six days of culture, Treg cells were collected and costained with Fluorescein isothiocyanate (FITC)-conjugated anti-mouse CD4 antibody (eBioscience, 11–0042-85) and PE-conjugated anti-mouse Foxp3 antibody (eBioscience, 12–4771-82).

CD4^+^ T cells were isolated from peripheral blood of healthy people by immunosorting with anti-CD4 microbeads (Stemcell, 19052), co-cultured with pDC (CAL-1) or not in six-well plate, all samples were in RPMI 1640 medium same as above, in addition for samples with human plasmacytoid dendritic cells (pDC) supplemented with 25 ng/mL interferon gamma (Peprotech, 300–02) and 5 μg/mL LPS (Sigma, L6143). Six days later, the cells were collected and contained with FITC-conjugated anti-human CD4 antibody (BD, 555346) and PE-conjugated anti-human Foxp3 antibody (eBioscience, 12–4776-42) to do the fluorescence-activated cell sorting (FACS) analysis with FACSCalibur (Becton Dickinson, Franklin Lakes, NJ).

### 2.4. Animal experiment

The experimental procedures of the animal study were proved by the Animal Care and Use Committee at Nankai University. Animal experiment was performed as previously described^20^. Six to eight weeks old female C57BL/6 mice were inoculated subcutaneously with 1 × 10^6^ B16F10 tumor cells into the left chest of each mouse. C57BL/6 mice bearing B16F10 tumors were treated orally once daily with 100 mg/kg IDO1 inhibitors (Roxyl-WL or 1-MT) were dissolved in 5% DMA, 47.5% propylene glycol, or vehicle. Tumor measurements were used using a digital vernier caliper and the volumes were determined using the following calculation: (short^2^) × long × 0.5. Inhibition rate of tumor growth was calculated using the following formula: 100 ×｛1−[(tumor volume_final_ −tumor volume_initial_) for Roxyl-WL-treated group]/[(tumor volume_final_ −tumor volume_initial_) for the vehicle-treated group]｝.

### 2.5. Western blot analysis

Western blot experiment was performed as previously reported^21^. The following antibodies were used: IDO1 (1:1000; sc-137012, Santa Cruz), Foxp3 (1:1000; ab20034, Abcam), and β-actin (1:5000; sc-47778, Santa Cruz).

### 2.6. High-performance liquid chromatography (HPLC) Kyn/Trp ratio

Kyn/Trp ratio was determined by measuring the concentrations in mice plasma using a Shimadzu Prominence-i LC-2030C 3 D HPLC system.

### 2.7. Immunohistochemistry analysis

The frozen sections were fixed in cold methanol for 15 min and then incubated with anti-IDO1 (1:100) or anti-Foxp3 (1:100) antibody overnight at 4 °C, followed by incubation with Alexa Fluor 549 goat anti-rat IgG or Alexa Fluor 488 goat anti-rabbit IgG, correspondingly at room temperature for 1 h. They were finally incubated with 4’,6-diamidino-2-phenylindole (DAPI; Sigma-Aldrich, St. Louis, MO) for nuclear counterstaining. Images were acquired by using a laser scanning confocal microscope (Leica, Wetzlar, Germany)^22^.

### 2.8. Statistical analysis

Statistical analysis of preliminary data was performed as previously described^20^.

## 3. Results and discussion

We performed virtual screening (VS) to find novel IDO1 inhibitors. We firstly performed molecular-docking-based VS two commercial libraries (Specs and ChemDiv) and our own library, which totally contained approximately 1,460,000 compounds. The compound database was first filtered by Lipinski properties (MW <500, ClogP <5, 0 < Rotational bonds <10, 0 < H-bond donor <5, 0 < H-bond acceptor <10) and led to about 550,000 compounds. The receptor structure for molecular docking was prepared from the crystal structure of IDO1 (PDB entry: 4PK5). The binding site was defined as a sphere containing the residues that stay within 8 Å from its original ligand, which was large enough to cover the catalytic site. The Charmm force field was assigned. All the docking calculations were carried out with the GOLD program^23^. We chose the top 15,000 compounds that were ranked by the “Goldscore” scoring function^24^. These compounds were further subjected to the next step screening.

As inherent limitations of each of screening techniques are not easily resolved, pharmacophore-based VS and docking-based VS combination in a hybrid protocol can help to mutually compensate for these limitations and capitalize on their mutual strengths^25^. In the pharmacophore-based VS approach, a pharmacophore hypothesis is taken as a template. Based on the crystal structure of IDO1/Amg-1 complex (PDB entry: 4PK5) as shown in Supplementary Figure S2, we found that the nitrogen of thiazolotriazole in Amg-1 was directly bound to the heme iron and that tolyl group located adjacent to Val130, Phe164, and Cys129. In addition, there is a hydrogen bond formed between the oxygen of dioxolane and Arg231. Therefore, we created a pharmacophore model manually which contained three features: hydrophobe, metal interaction point, and acceptor feature (Supplementary Figure S2). The 3 D database was built from the 15,000 top ranking compounds screened by molecular docking. Then pharmacophore-model-based VS was performed against the 15,000 compounds and 232 molecules match the condition.

**Figure 1.**
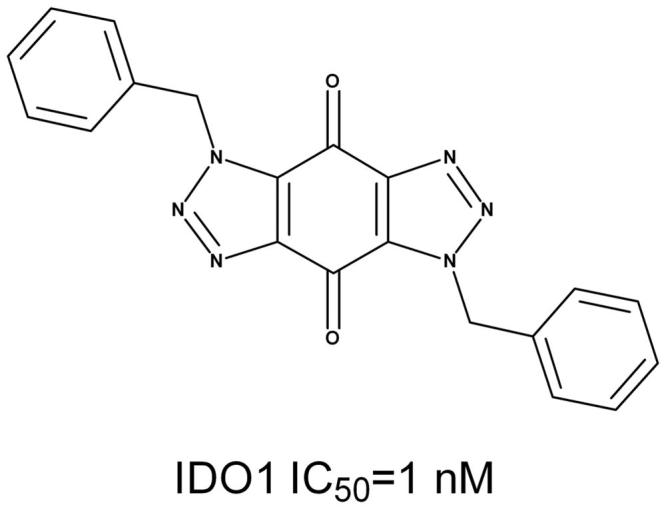
The chemical structure and the IC_50_ value of Roxyl-WL against IDO1.

**Figure 2.**
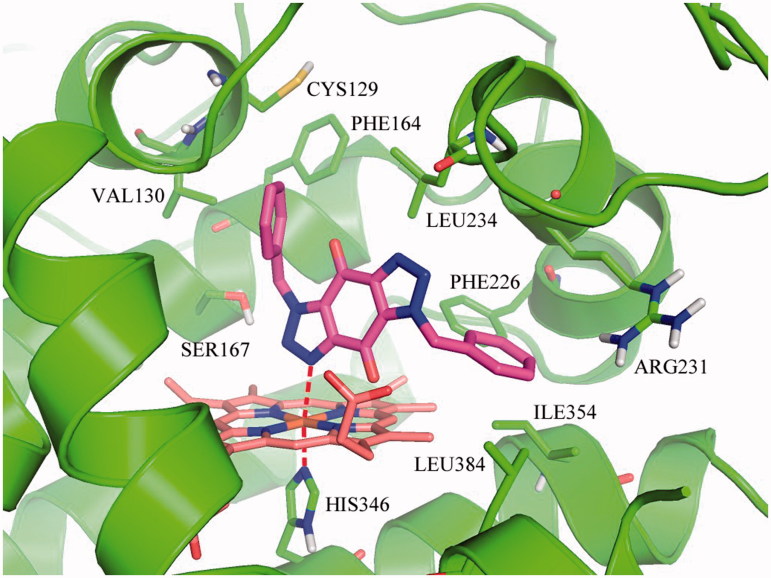
Predicted binding model of compound Roxyl-WL in the active pocket of IDO1. Compound Roxyl-WL is colored in magenta, Fe(II) is in brown and residues of IDO1 are in green. Coordination bonds are shown in red dashed lines.

On basis of the binding conformation of the 232 compounds in IDO1 active pocket, 30 compounds (Supplementary Figure S3) were finally selected for bioassays. The IDO1 enzymatic inhibition assay was served by BPS Bioscience Inc., San Diego, CA. The majority of the tested compounds showed low or no effects in the *invitro* assay except Compound **1** and **2**. (Supplementary Table S1). Further structural optimization and analysis are carried out on the most active Compound **2**. As Compound **2** contains a 1*H*-benzo[d] [1,2,3]triazole-4,7-dione core, 18 structure-like compounds were synthesised or purchased (Supplementary Figure S4). The compounds information and synthesis routes are provided in the Supplemental material. The enzyme activity of these compounds against IDO1 at the concentration of 10 μM is shown in Supplementary Table S2. Compound Roxyl-11 (Roxyl-WL) showed outstanding inhibition rate of 100% against IDO1 at 10 μM. The IC_50_ value of Roxyl-WL were further examined and it showed excellent inhibitory activity against IDO1 with an IC_50_ value of 1 nM (Figure 1). Carbon-13 (C13) nuclear magnetic resonance and purity scan of Roxyl-WL are in the Supplementary material. Molecular docking was used again to predict the binding model of compound Roxyl-WL in the active pocket of IDO as shown in Figure 2. Roxyl-WL suitably resides in the active pocket of IDO1 with the nitrogen of trinitrogenazole pointing towards Fe (II) through forming a coordination interaction between the nitrogen and Fe (II). Molecular docking was also used to predict the binding model between compound 1-MT and IDO1 (Supplementary Figure S5). From Supplementary Figure S5, we can see that there is a hydrogen bond formed between the amino-group and the ferroheme, but the compound only occupies half of the active pocket. So it is obvious that they don’t match as good as the binding mode between Roxyl-WL and IDO1.

Roxyl-WL containing the quinone substructure has been flagged as PAINS-liable compounds as detected by the pan assay interference compounds (PAINS)filters^26^. To further investigate whether Roxyl-WL is promiscuous with activity to multiple unrelated biological targets, kinase inhibition profiling assays of Roxyl-WL were carried out against a series of 337 kinases through the Eurofins kinase profiling. All of the 337 kinases present diverse classes of proteins and have different biological functions. Roxyl-WL exhibited no activity to any of the protein or receptors (Supplementary Table S3). As mentioned above, Roxyl-WL is not a PAINS candidate, but a highly potent and selective IDO1inhibitor.

**Figure 3.**
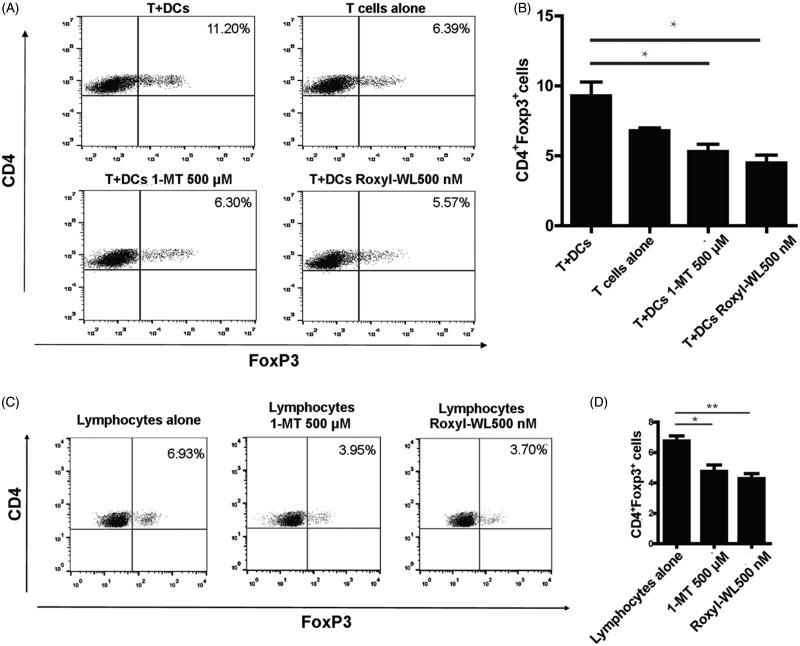
IDO1 inhibition with Roxyl-WL reduced the conversion of Treg-like cells *in vitro*. (A,B) Human CD4^+^ T cells were cultured in the presence of pDCs 500 nM and Roxyl-WL or 500 μM 1-MT for 6 days. (C,D) Lymphocytes from the spleen of normal C57BL/6 mice were cultured with 500 nM Roxyl-WL or 500 μM 1-MT for six days. Cells were costained for CD4 and Foxp3 expression. (A,C) A representative plot of FACS analysis is presented. (B,D) Average values of three independent experiments are shown in the graph. Error bars represent SD.

IDO1 promoting the conversion of naive CD4^+^ T cell to Treg cell phenotype has recently been described^27^. An increase of Treg cell activity is associated with tumor growth. Treg cell depletion enhances antitumor immune responses^13^^,^^28^. Inhibition of IDO1 could reduce the development and activation of Treg cells^29^. As reported previously, DCs have the ability to promote Treg cell conversion, while IDO1 inhibitors have the potential to reverse this effect. IDO1 inhibitors could stimulate producing IFNγ from T cells when cocultured with DCs to effect on the proliferation of T cells^30^. Thus, it was interested to detect whether our compound Roxyl-WL could influence the Treg cell conversion. We extracted CD4^+^ T lymphocytes from peripheral blood of healthy person by immunosorting with anti-CD4 microbeads, cocultured with pDCs (CAL-1) in the presence or absence of Roxyl-WL. The typical IDO1 inhibitor 1-MT was used as a positive control with a concentration of 500 μM according to the reference^13^. After six days of incubation, we did the FACS analysis to test the amount of Tregs which costained by the CD4 and Foxp3 antibody. As shown in Figure 3(A,B), coculture of naive CD4^+^ T cells with DCs resulted in an approximately two-fold increase in the number of CD4^+^ Treg cells. Addition of our compound Roxyl-WL with the concentration 500 nM to the cultures reversed this effect, as did treatment with 500 μM 1-MT.

Using the same method, we extracted lymphocytes from the spleen of normal C57BL/6 mice and separately cultured with Roxyl-WL with a fixed concentration of 500 nM. As shown in Figure 3(C,D), we found that Roxyl-WL showed better capability in reducing Tregs compared with the control group. From immune perspective, Roxyl-WL can reduce the number of Foxp^3+^ Treg cells *in vitro*, which could indeed improve immune ability as to achieve the purpose of antitumor.

**Figure 4.**
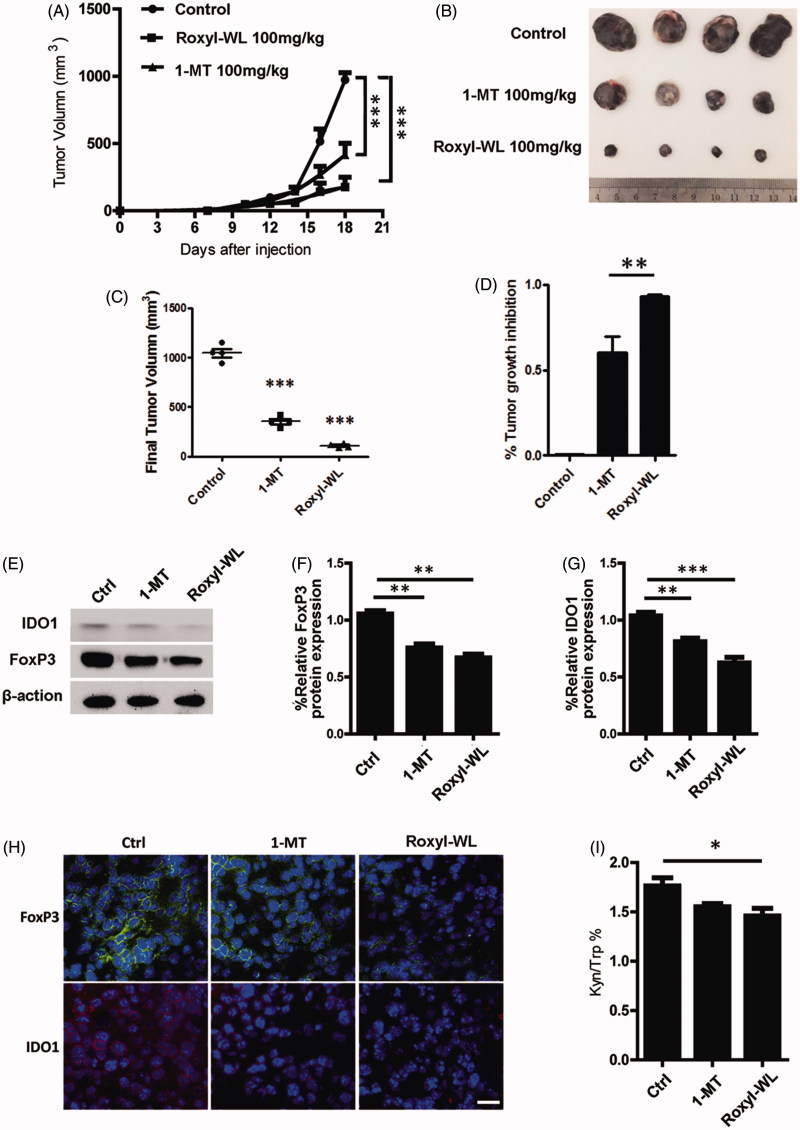
IDO1 inhibition with Roxyl-WL suppressed tumour growth with immune efficacy in B16F10 tumour-bearing mice model. (A) Single-agent Roxyl-WL suppressed tumour growth effectively. (B) Representative photograph of excised tumours (*n* = 4) for each treatment group at day 10. (C) Volumes of final excised tumours for each treatment group at day 10. (D) Tumour growth inhibition (%) for each treatment group. (E) Roxyl-WL treatment led to decreased IDO1 and Foxp3 protein expression in the tumour tissues. (F,G) Average values of three independent experiments are shown in the graph E. Error bars represent standard deviation. (H) Roxyl-WL treatment led to decreased IDO1 and Foxp3 expression by immunofluorescence analysis. Scale bar = 30 μm. (I) Kyn/Trp ratios were calculated after 10 days of treatment. Plasma was harvested and Kyn and Trp levels were determined by HPLC.

The activity of IDO1 inhibitors suppressing Tregs has been shown *in vitro*. To investigate whether Roxyl-WL could similarly reverse immune escape *in vivo*, we treated C57BL/6 mice bearing B16F10 tumor orally with Roxyl-WL or 1-MT for 10 days. We carried out preliminary *in vivo* studies to determine the maximum tolerated dose of compounds in mice. Administration of compounds by oral gavage with different doses (50, 100, and 150 mg/kg) were monitored for 12 consecutive days, 150 mg/kg dose showed certain toxicity. In the meantime, according to the reference reported before, 100 mg/kg was the appropriate dose^13^^,^^21^^,^^31^. Therefore, we selected 100 mg/kg as the final dose. Roxyl-WL group showed greater ability of tumor suppression obviously (Figure 4(A)). Mice treated with Roxyl-WL had a significant reduction of the tumor size was compared with 1-MT group (Figure 4(B,C)). Tumor growth inhibitions of 91.5% were observed at doses of 100 mg/kg in Roxyl-WL-treated group. In contrast, 1-MT as the positive control was less potent (60.4%) than Roxyl-WL at the same dose (Figure 4(D)). These results suggest that Roxyl-WL has the therapeutic potential to be a superior candidate as a single drug for tumor treatment.

Previous studies reported that IDO1 inhibitors could affect the IDO1 level *in vivo*. To further investigate the IDO1 expression *in vivo*, we extracted the protein from mice tumor tissues after 10 days of IDO1 inhibitors treatment. We found that IDO1 expression in Roxyl-WL treated tumors was lower than that of 1-MT group (Figure 4(E,G)). Immunofluorescence experiment also demonstrated this result (Figure 4(H)). It does illustrate that Roxyl-WL play a role on the IDO1 expression of tumor *in vivo*.

Foxp3 is not only a key intracellular marker but also a crucial developmental and functional factor for CD4^+^ Tregs^21^. In animal models, as shown in Figure 4(E,F), we also proved that Foxp3 expression was significantly reduced in Roxyl-WL treated tumors and less reduced in 1-MT treated tumors. Similar results were obtained by immunofluorescence analysis (Figure 4(H)). The data demonstrated that Roxyl-WL as a highly potent IDO1 inhibitor can significantly reduce the number of Foxp^3+^ Tregs in B16F10 tumor-bearing mice.

Trp degradation and Kyn production are the indicators of IDO1 activity. IDO1 enzyme activity can be reflected by the Kyn/Trp ratio. Therefore, in our *in vivo* experiment, we harvested the plasma from B16F10 tumor-bearing C57BL/6 mice to examine Kyn/Trp ratio through using the HPLC method. As shown in Figure 4(I), the Kyn/Trp ratio of the Roxyl-WL group decreased more significantly than other groups. It means that Roxyl-WL relative 1-MT inhibits IDO1 enzyme activity more effectively.

## 4. Conclusion

In summary, molecular docking and pharmacophore-based VS finally led to the discovery of compound Roxyl-WL (Supplementary Figure S6). Roxyl-WL exhibits excellent IDO1 enzyme activity (IC_50_ =1 nM), many fold over the IDO1 inhibitors as previously reported. Roxyl-WL displayed outstanding kinase spectrum selectivity with no activity out of the 337 protein kinases. *In vitro*, the removal of IDO1 enzymatic activity by Roxyl-WL can effectively suppress the conversion of naive CD4^+^ T cell to Treg cell phenotype. When administrated to the B16F10 tumor-bearing mice, Roxyl-WL can inhibit IDO1 activity, suppress the tumor growth, reduce the number of Foxp^3+^ Tregs, and decrease the Kyn/Trp ratio evidently. Taken together, Roxyl-WL represents a novel IDO1 inhibitor against diseases characterized by immune suppression and is a immunotherapeutic agent, which may help to resist the immune tolerance of the tumor microenvironment.

## Supplementary Material

Supplemental Material
