# Supplementary material for: A highly potent and selective inhibitor Roxyl-WL targeting IDO1 promotes immune response against melanoma
Source: J Enzyme Inhib Med Chem. 2018 Jun 22;33(1):1089–94. doi: 10.1080/14756366.2018.1471688 (PMC6022239; doi:10.1080/14756366.2018.1471688)
Supplement: Supplemental Material [file IENZ_A_1471688_SM1990.pdf]

Supporting Information

A Highly Potent and Selective Inhibitor Roxyl-WL Targeting IDO1 Promotes Immune Response against Melanoma

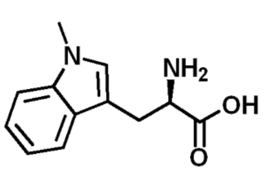

NLG-8189 (1-MT)

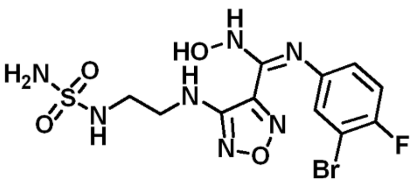

INCB024360 (Epacadostat)

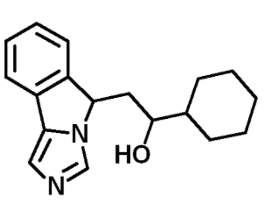

NLG919

Figure S1. Chemical Structures of representative IDO1 Inhibitors.

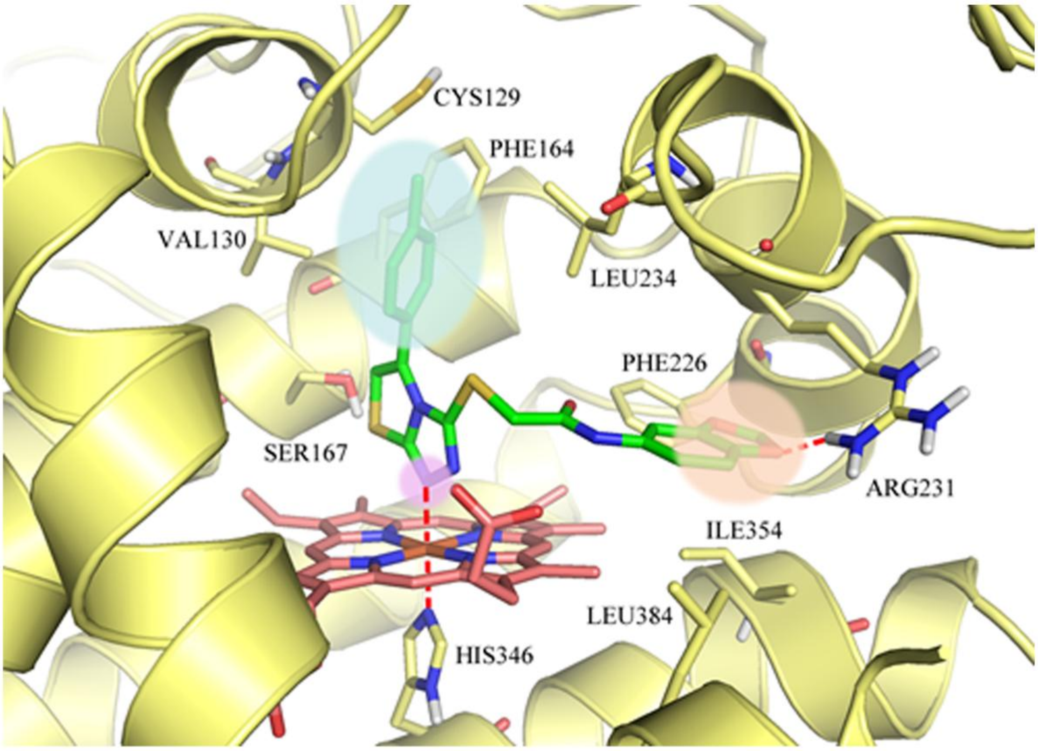

Figure S2. The pharmacophore features we defined based on the complex structure between IDO1 and its ligand (PDB entry: 4PK5). Hydrophobe, metal interaction point and acceptor feature are shown in turquois, purple and orange respectively.

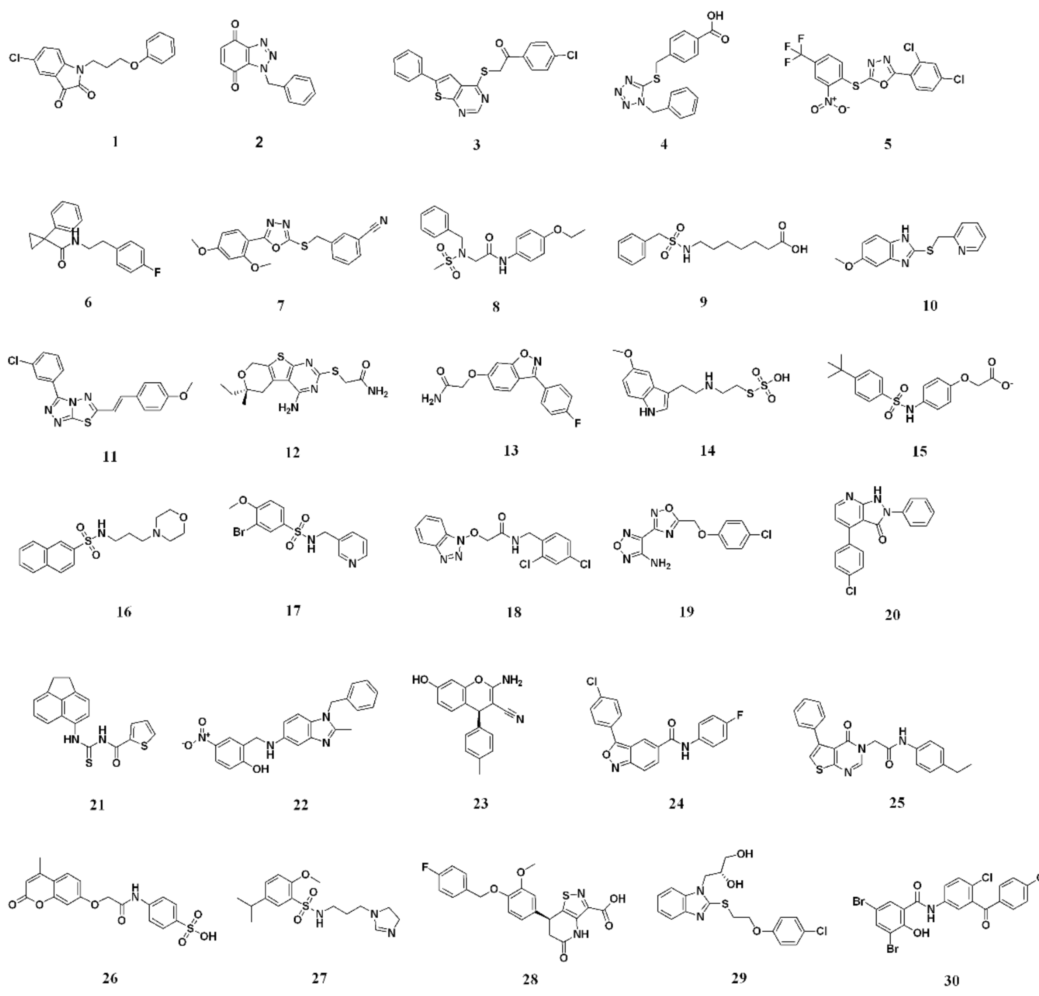

Figure S3. The chemical structures of 30 hit compounds by molecular docking and pharmacophore modeling.

Table S1. Enzymatic Inhibition of IDO1 at 10μM of 30 hit compounds.[a]

Comp.	Inhibition% at 10 μM[a]	Comp.	Inhibition% at 10μM[a]	Comp.	Inhibition% at 10 μM[a]
**1**	33	**11**	0	**21**	13
**2**	72	**12**	5	**22**	12
**3**	12	**13**	6	**23**	15
**4**	5	**14**	6	**24**	11
**5**	5	**15**	7	**25**	14
**6**	4	**16**	6	**26**	19
**7**	8	**17**	5	**27**	19
**8**	2	**18**	2	**28**	10
**9**	2	**19**	5	**29**	12
**10**	6	**20**	17	**30**	16

[a] All assays were conducted in duplicate.

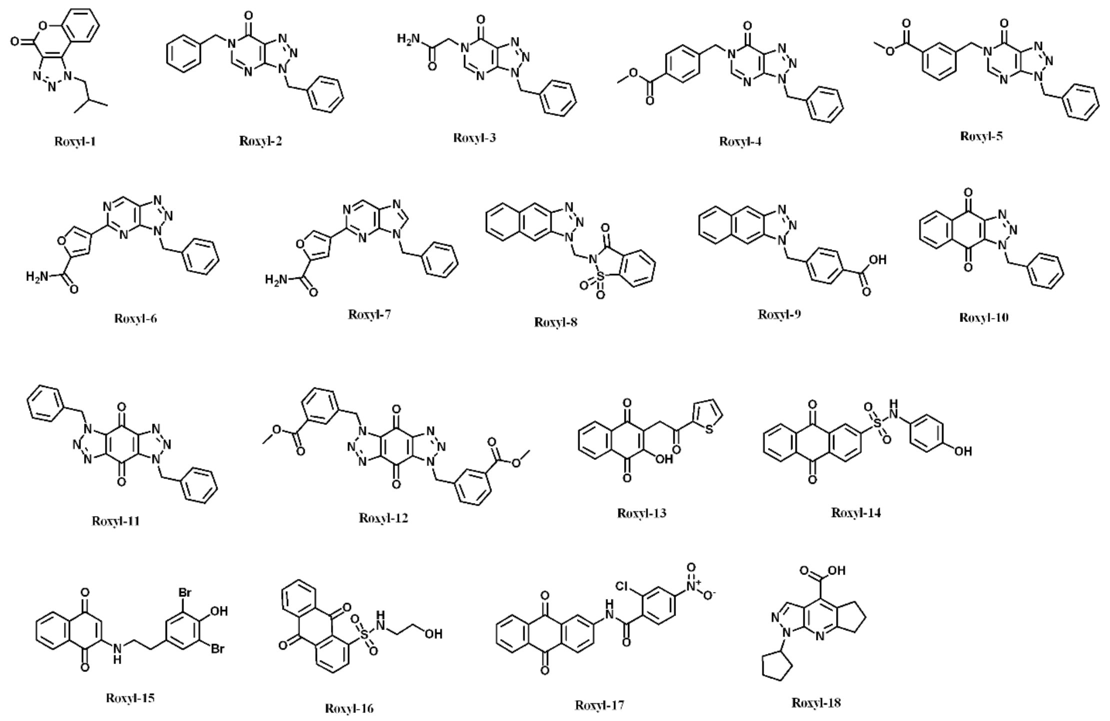

Figure S4. 18 structure-like compounds based on compound 2 with 1*H*-benzo[d][1,2,3]triazole-4,7-dione core.

Table S2. Enzymatic Inhibition of IDO1 at 10μM of 18 compounds. [a]

Comp.	Inhibition% at 10 μM	Comp.	Inhibition% at 10 μM
Roxyl-1	1	Roxyl-10	75
Roxyl-2	12	Roxyl-11	100
Roxyl-3	0	Roxyl-12	76
Roxyl-4	16	Roxyl-13	31
Roxyl-5	9	Roxyl-14	48
Roxyl-6	7	Roxyl-15	45
Roxyl-7	6	Roxyl-16	60
Roxyl-8	21	Roxyl-17	4
Roxyl-9	8	Roxyl-18	7

[a] All assays were conducted in duplicate.

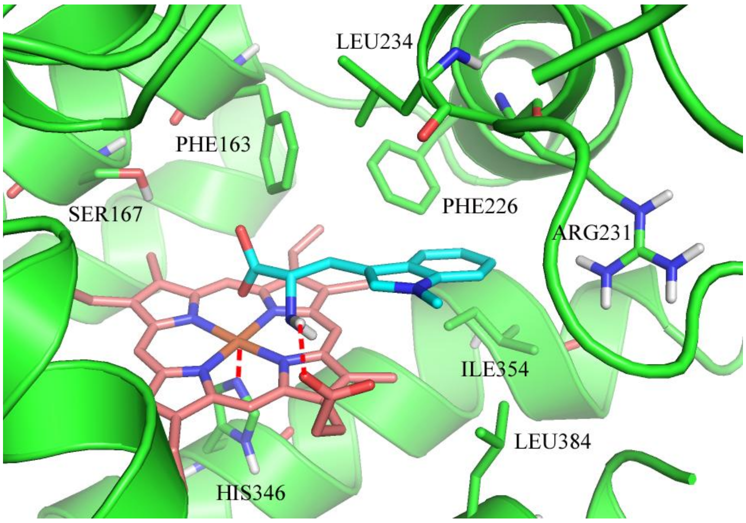

Figure S5. Predicted binding model of compound 1-MT in the active pocket of IDO1. Compound 1-MT is colored in cyan. Fe(II) is in brown and residues of IDO1 are in green. The hydrogen bond and the coordination bond are shown in red dashed line.

Table S3. Kinase Profiling Results of compound Roxyl- WL[a]

Kinase	Inhibition % at 10 μM	Kinase	Inhibition % at 10 μM
Abl(h)	42	MAPKAP-K2(h)	-5
Abl (H396P) (h)	25	MAPKAP-K3(h)	3
Abl (M351T)(h)	19	MEK1(h)	25
Abl (Q252H) (h)	33	MARK1(h)	-2
Abl(T315I)(h)	40	MARK4(h)	1
Abl(Y253F)(h)	38	MEKK2(h)	-13
ACK1(h)	13	MELK(h)	21
ALK(h)	-23	Mer(h)	5
ALK1(h)	10	Met(h)	1
ALK2(h)	9	Met(D1246H)(h)	-13
ALK4(h)	8	Met(D1246N)(h)	-5
ALK6(h)	2	Met(M1268T)(h)	12
Arg(h)	-6	Met(Y1248C)(h)	-1
AMPKα1(h)	3	Met(Y1248D)(h)	1
AMPKα2(h)	-9	Met(Y1248H)(h)	6
A-Raf(h)	19	MINK(h)	1
ARK5(h)	5	MKK4(m)	14
ASK1(h)	-12	MKK6(h)	-2
Aurora-A(h)	3	MKK7β(h)	-6
Aurora-B(h)	39	MLCK(h)	2
Aurora-C(h)	2	MLK1(h)	12
Axl(h)	-7	MLK2(h)	-1
Blk(h)	4	Mnk2(h)	3
Bmx(h)	-40	MOK(h)	2
BRK(h)	7	MRCKα(h)	-1
BrSK1(h)	11	MRCKβ(h)	-12
BrSK2(h)	18	MSK1(h)	-5
BTK(h)	1	MSK2(h)	14
BTK(R28H)(h)	2	MSSK1(h)	-7
B-Raf(h)	31	MST1(h)	22
B-Raf(V599E)(h)	24	MST2(h)	-4
CaMKI(h)	13	MST3(h)	-7
CaMKIγ(h)	11	MST4(h)	-1
CaMKIIα(h)	-5	mTOR(h)	-4
CaMKIIβ(h)	0	mTOR/FKBP12(h)	-1
CaMKIIγ(h)	3	MuSK(h)	6
CaMKIδ(h)	23	MYLK2(h)	-3
CaMKIIδ(h)	8	NEK2(h)	-1

CaMKIV(h)	-8	NEK3(h)	-7
CaMKK2(h)	3	NEK6(h)	2
CDK1/cyclinB(h)	0	NEK7(h)	3
CDK2/cyclinA(h)	20	NEK9(h)	-3
CDK2/cyclinE(h)	1	NIM1(h)	-12
CDK3/cyclinE(h)	1	NEK11(h)	-9
CDK4/cyclinD3(h)	0	NLK(h)	-10
CDK5/p25(h)	2	NUAK2(h)	-2
CDK5/p35(h)	-1	p70S6K(h)	8
CDK6/cyclinD3(h)	10	PAK1(h)	4
CDK7/cyclinH/MAT1(h)	9	PAK2(h)	-15
CDK9/cyclin T1(h)	5	PAK4(h)	0
ChaK1(h)	-1	PAK5(h)	23
CHK1(h)	-1	PAK6(h)	15
CHK2(h)	10	PAR-1Bα(h)	-2
CHK2(I157T)(h)	9	PASK(h)	15
CHK2(R145W)(h)	5	PEK(h)	1
CK1γ1(h)	-6	PDGFRα(h)	-16
CK1γ2(h)	18	PDGFRα(D842V)(h)	5
CK1γ3(h)	5	PDGFRα(V561D)(h)	-9
CK1δ(h)	14	PDGFRβ(h)	-4
CK1(y)	-12	PDK1(h)	-2
CK2(h)	-10	PhKγ2(h)	8
CK2α2(h)	-9	Pim-1(h)	5
CLK1(h)	18	Pim-2(h)	4
CLK2(h)	28	Pim-3(h)	8
CLK3(h)	-8	PKA(h)	7
CLK4(h)	13	PKAcβ(h)	29
cKit(h)	42	PKBα(h)	3
cKit(D816V)(h)	-11	PKBβ(h)	16
cKit(D816H)(h)	2	PKBγ(h)	12
cKit(V560G)(h)	0	PKCα(h)	15
cKit(V654A)(h)	-6	PKCβI(h)	5
CSK(h)	14	PKCβII(h)	5
c-RAF(h)	27	PKCγ(h)	2
cSRC(h)	17	PKCδ(h)	-6
DAPK1(h)	-6	PKCε(h)	2
DAPK2(h)	-6	PKCη(h)	-3
DCAMKL2(h)	7	PKCι(h)	5
DCAMKL3(h)	5	PKCμ(h)	55
DDR1(h)	-3	PKCθ(h)	9
DDR2(h)	-1	PKCζ(h)	-9
DMPK(h)	-11	PKD2(h)	0

DRAK1(h)	16	PKG1α(h)	6
DYRK1A(h)	8	PKG1β(h)	6
DYRK1B(h)	15	PKR(h)	-11
DYRK2(h)	-8	Plk1(h)	1
DYRK3(h)	-17	Plk3(h)	6
eEF-2K(h)	13	PRAK(h)	-4
EGFR(h)	6	PRK2(h)	5
EGFR(L858R)(h)	7	PrKX(h)	9
EGFR(L861Q)(h)	11	PTK5(h)	23
EGFR(T790M)(h)	5	Pyk2(h)	12
EGFR(T790M,L858R)(h)	3	Ret(h)	24
EphA1(h)	-10	Ret (V804L)(h)	-23
EphA2(h)	5	Ret(V804M)(h)	-5
EphA3(h)	-26	RIPK2(h)	-9
EphA4(h)	-1	ROCK-I(h)	-7
EphA5(h)	2	ROCK-II(h)	-6
EphA7(h)	-4	Ron(h)	-10
EphA8(h)	-5	Ros(h)	6
EphB2(h)	-43	Rse(h)	4
EphB1(h)	-44	Rsk1(h)	13
EphB3(h)	-33	Rsk2(h)	15
EphB4(h)	4	Rsk3(h)	-4
ErbB2(h)	3	Rsk4(h)	16
ErbB4(h)	6	SAPK2a(h)	17
FAK(h)	23	SAPK2a(T106M)(h)	-2
Fer(h)	-6	SAPK2b(h)	8
Fes(h)	14	SAPK3(h)	2
FGFR1(h)	13	SAPK4(h)	-4
FGFR1(V561M)(h)	11	SGK(h)	35
FGFR2(h)	6	SGK2(h)	7
FGFR2(N549H)(h)	3	SGK3(h)	23
FGFR3(h)	16	SIK(h)	1
FGFR4(h)	4	Snk(h)	7
Fgr(h)	7	SNRK(h)	17
Flt1(h)	31	Src(1-530)(h)	17
Flt3(D835Y)(h)	12	Src(T341M)(h)	-4
Flt3(h)	24	SRPK1(h)	6
Flt4(h)	13	SRPK2(h)	3
Fms(h)	0	STK25(h)	-6
Fms(Y969C)(h)	4	STK33(h)	-4
Fyn(h)	14	Syk(h)	-8
GCK(h)	4	TAK1(h)	1
GCN2(h)	12	TAO1(h)	0

GRK1(h)	8	TAO2(h)	-7
GRK2(h)	3	TAO3(h)	16
GRK5(h)	1	TBK1(h)	-4
GRK6(h)	4	Tec(h) activated	8
GRK7(h)	-3	TGFBR1(h)	0
GSK3α(h)	7	Tie2 (h)	8
GSK3β(h)	34	Tie2(R849W)(h)	3
Haspin(h)	29	Tie2(Y897S)(h)	-9
Hck(h)	-4	TLK1(h)	0
Hck(h) activated	4	TLK2(h)	5
HIPK1(h)	-2	TrkA(h)	10
HIPK2(h)	-13	TrkB(h)	-33
HIPK3(h)	0	TrkC(h)	20
HIPK4(h)	-10	TSSK1(h)	10
HPK1(h)	-6	TSSK2(h)	11
IGF-1R(h)	-8	Txk(h)	2
IGF-1R(h), activated	-5	TYK2(h)	3
IKKα(h)	3	ULK1(h)	2
IKKβ(h)	-4	ULK2(h)	1
IKKε(h)	0	ULK3(h)	-3
IR(h)	5	Wee1(h)	-1
IR(h), activated	-4	WNK2(h)	4
IRE1(h)	-1	WNK3(h)	2
IRR(h)	0	VRK2(h)	0
IRAK1(h)	4	Yes(h)	7
IRAK4(h)	-12	ZAP-70(h)	-12
Itk(h)	5	ZIPK(h)	13
JAK1(h)	-7	ATM(h)	38
JAK2(h)	1	ATR/ATRIP(h)	50
JAK3(h)	24	DNA-PK(h)	41
JNK1α1(h)	-16	PI3 Kinase (p110 /p85)(h)	-1
JNK2α2(h)	5	PI3 Kinase (p120)(h)	3
JNK3(h)	2	PI3 Kinase (p110 /p85)(h)	6
KDR(h)	-3	PI3 Kinase (p110 /p85)(m)	-2
Lck(h)	9	PI3 Kinase (p110 /p85)(h)	1
Lck(h) activated	-18	PI3 Kinase (p110a(E542K)/p85a)(h)	-1
LIMK1(h)	4	PI3 Kinase (p110a(H1047R)/p85a)(h)	1
LKB1(h)	-2	PI3 Kinase (p110a(E545K)/p85a)(h)	-1
LOK(h)	8	PI3 Kinase (p110a/p65a)(h)	-2
Lyn(h)	13	PI3KC2a(h)	-5
LRRK2(h)	23	PI3KC2g(h)	-3
LTK(h)	-5	PIP4K2a(h)	0
MAPK1(h)	-22	PIP5K1a(h)	-4

MAPK2(h)	-14	PIP5K1g(h)	-1
MAP4K5(h)	2		

[a]Values were determined using Kinase Profiler by Eurofins. The data represent the mean values of two independent experiments

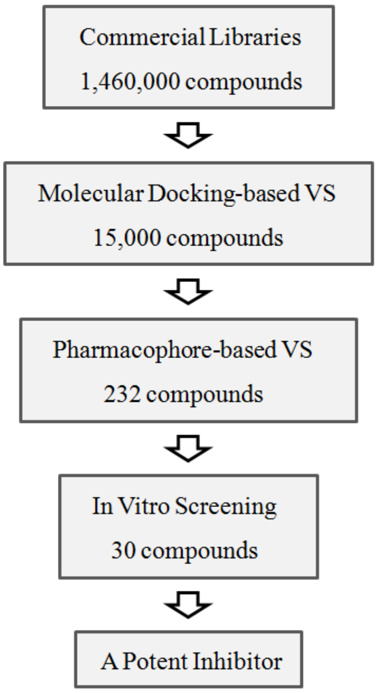

Figure S6. Flow chart for the discovery of the IDO1 inhibitor (Roxyl-WL).

Chemistry

Roxyl-1 (cas: 431882-53-0) was purchased from Specs (Specs, Inc. Zoetermeer, The Netherlands).

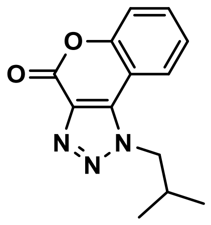

Roxyl-1

3,6-dibenzyl-3,6-dihydro-7H-[1,2,3]triazolo[4,5-d]pyrimidin-7-one (**Roxyl-2**)

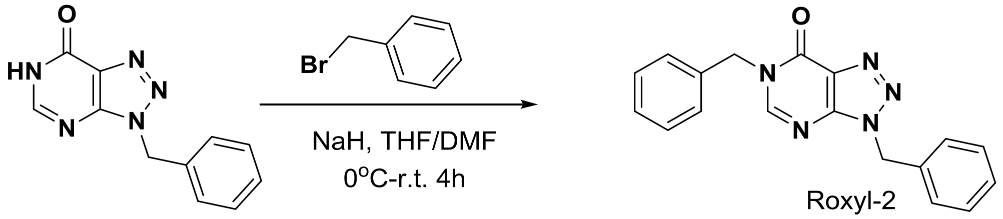

Roxyl-2 (cas :428849-26-7) was prepared by 3-benzyl-3,6-dihydro-7H-[1,2,3]triazolo[4,5-d]pyrimidin-7-one (cas: 21324-31-2) with benzyl bromide.

To a solution of 3-benzyl-3,6-dihydro-7H-[1,2,3]triazolo[4,5-d]pyrimidin-7-one (2.27 g, 10 mmol, 1 equiv.) in dry DMF (20 mL) at 0 °C was successively added NaH (40 mg, 11 mmol, 1.1 equiv, 60%). After stirring for 1h, benzyl bromide (1.88 g, 11 mmol, 1.1 equiv) in dry THF (20 mL) was dropwise added. After added, the solution was removed to r.t., and stirring for 3h. Water (5 mL) was added and the reaction mixture was partitioned between EA (250 mL) and water (100 mL). After separation, the organic layers were washed once with water and brine and dried over MgSO4. Purification by silica gel column chromatography to obtain 2.279 g (72%) of **Roxyl-2** as a white solid. ^{1}H NMR (400 MHz, DMSO-d_6) δ 8.83 (s, 1H), 7.43 – 7.24 (m, 10H), 5.77 (s, 2H), 5.25 (s, 2H).

Roxyl-3 (CAS: 863018-21-7) was purchased from Specs (Specs, Inc. Zoetermeer, The Netherlands).

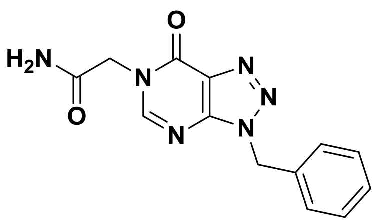

Roxyl-3

methyl 4-((3-benzyl-7-oxo-3,7-dihydro-6H-[1,2,3]triazolo[4,5-d]pyrimidin-6-yl)methyl)benzoate (**Roxyl-4**)

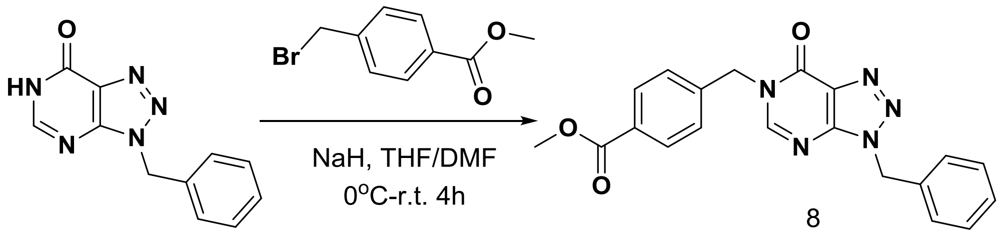

Roxyl-4 was prepared by 3-benzyl-3,6-dihydro-7H-[1,2,3]triazolo[4,5-d]pyrimidin-7-one (cas: 21324-31-2) with methyl 4-(bromomethyl)benzoate following the procedure of compound **Roxyl-2**. White solid, 73% yeild. 1H NMR (400 MHz, DMSO-d6) δ 8.82 (s, 1H), 8.02 – 7.83 (m, 2H), 7.54 – 7.45 (m, 2H), 7.41 – 7.29 (m, 5H), 5.78 (s, 2H), 5.32 (s, 2H), 3.83 (s, 3H). 13C NMR (101 MHz, DMSO) δ 165.85, 154.61, 152.64, 147.98, 141.75, 135.19, 129.41, 128.90, 128.76, 128.16, 127.88, 127.71, 127.40, 52.12, 49.78, 48.64.

methyl 3-((3-benzyl-7-oxo-3,7-dihydro-6H-[1,2,3]triazolo[4,5-d]pyrimidin-6-yl)methyl)benzoate (**Roxyl-5**)

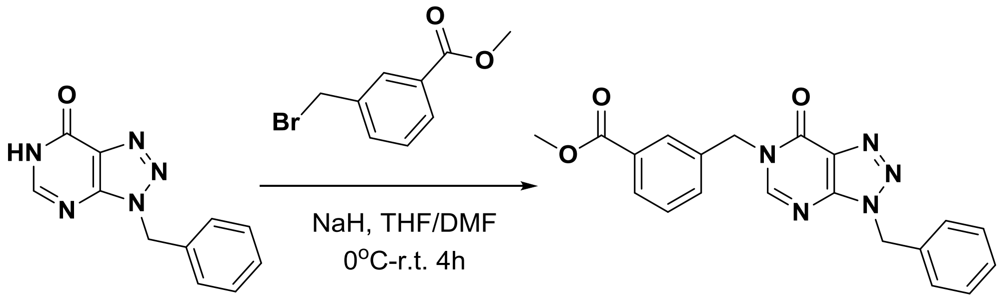

Roxyl-5

Roxyl-5 was prepared by 3-benzyl-3,6-dihydro-7H-[1,2,3]triazolo[4,5-d]pyrimidin-7-one (cas: 21324-31-2) with methyl 3-(bromomethyl)benzoate following the procedure of compound **Roxyl-2**. White solid, 78% yeild. ^{1}H NMR (400 MHz, DMSO-d_6) δ 8.89 (s, 1H), 8.02 (t, J = 1.8 Hz, 1H), 7.88 (d, J = 7.7 Hz, 1H), 7.68 (dt, J = 7.8, 1.5 Hz, 1H), 7.50 (t, J = 7.7 Hz, 1H), 7.46 – 7.27 (m, 5H), 5.77 (s, 2H), 5.32 (s, 2H), 3.84 (s, 3H). ^{13}C NMR (101 MHz, DMSO) δ 165.89, 154.63, 152.54, 147.93, 137.10, 135.18, 132.79, 129.89, 129.11, 129.05, 128.74, 128.62, 128.56, 128.14, 127.85, 52.19, 49.78, 48.57.

4-(3-benzyl-3H-[1,2,3]triazolo[4,5-d]pyrimidin-5-yl)furan-2-carboxamide (**Roxyl-6**) and 4-(9-benzyl-9H-purin-2-yl)furan-2-carboxamide (**Roxyl-7**)

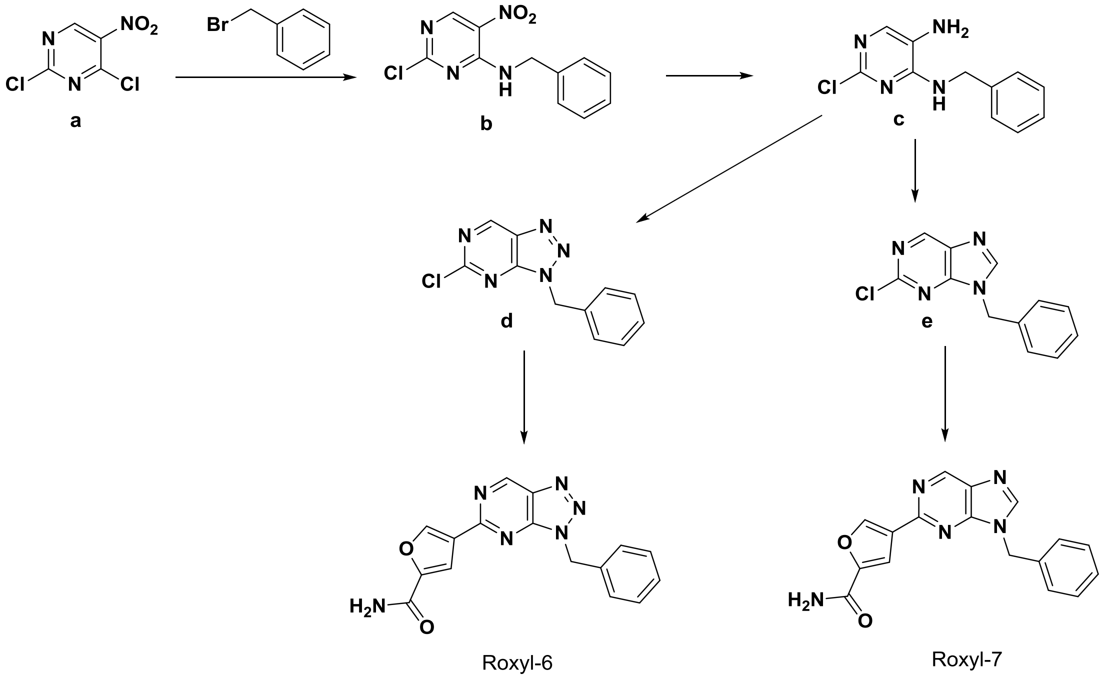

N-benzyl-2-chloro-5-nitropyrimidin-4-amine (**b**)

To a solution of compound **a** (9.7 g, 50 mmol, 1 equiv) in dry EA (100 mL) at -40 °C was successively added DIEA (6.47 g, 50 mmol, 1 equiv). After stirring for 5 min, benzylamine (5.36 g, 50 mmol, 1 equiv) in dry EA (100 mL) was dropwise added into the solution. After added, the solution was removed to r.t., and stirring for overnight. Water (200 mL) was added and the reaction mixture was partitioned between EA (200 mL). After separation, the organic layers were washed once with citrate solution, water and brine and dried over MgSO4. After evaporation of the volatiles, 13.34 g (99%) of

compound **b** was obtain as a yellow solid. ^{1}H NMR (400 MHz, Chloroform-*d*) δ 9.07 (s, 1H), 8.66 (s, 1H), 7.44 – 7.32 (m, 5H), 4.85 (d, *J* = 5.7 Hz, 2H).

N4-benzyl-2-chloropyrimidine-4,5-diamine (**d**)

To a solution of compound **b** (6.62 g, 25 mmol) in acetic acid (50 mL) at 0 °C was successively added Fe power (10 g, 179 mmol). After added, the solution was removed to 80 °C, and stirring for 3h. The crude solution was evaporated to dryness and neutralized by addition of $NaHCO_3$ solution (100 mL). The water phase was extracted with ethyl acetate. The combined organic layer was dried ($MgSO_4$), and after evaporation of the volatiles, the resulting compound was used directly for the next step. Sodium nitrite (2.07g, 30 mmol) in water (10 mL) was added to the solution of 10b in 40 mL of con. HCl at 0 °C, and the organic layers the reaction mixture was removed to r.t., and stirring for 3h. The solvent was extracted with ethyl acetate, and washed once with water and brine and dried over MgSO4. After evaporation of the volatiles, the resulting compound purification by silica gel column chromatography to obtain 5.16 g (84%) of compound **d** as a yellow solid. ^{1}H NMR (400 MHz, Chloroform-*d*) δ 9.39 (s, 1H), 7.53 – 7.44 (m, 2H), 7.44 – 7.32 (m, 3H), 5.85 (s, 2H).

4-(3-benzyl-3H-[1,2,3]triazolo[4,5-d]pyrimidin-5-yl)furan-2-carboxamide **(Roxyl-6)**

To a suspension of d (2 mmol, 1 equiv) in 20 mL 1,4-dioxane was added compound **4-(4,4,5,5-tetramethyl-1,3,2-dioxaborolan-2-yl)furan-2-carboxamide** (3 mmol, 1 equiv), Pd(dppf)Cl_2 (0.1 mmol, 0.05 equiv), DIEA (4 mmol, 2equiv) and water (5 mL) and the flask was purged with N_2. Then the flask was sealed and the mixture was heated for 12 h at 100 °C. The reaction was cooled to room temperature, the solvent was removed under reduced pressure, and the residue was purified by silica gel column chromatography to obtain **Roxyl-6**, as yellow solid. 79% yield. ^{1}H NMR (400 MHz, DMSO-d_6) δ 9.74 (s, 1H), 8.70 (s, 1H), 8.06 (s, 1H), 7.80 (s, 1H), 7.61 (s, 1H), 7.43 (d, *J* = 6.9 Hz, 2H), 7.41 – 7.28 (m, 3H), 5.94 (s, 2H). ^{13}C NMR (101 MHz, DMSO) δ 159.41, 158.23, 152.98, 149.78, 149.69, 148.11, 135.67, 134.38, 129.32, 128.74, 128.61, 127.98, 112.85, 50.15.

9-benzyl-2-chloro-9H-purine (**e**)

To a solution of compound **b** (6.62 g, 25 mmol) in acetic acid (50 mL) at 0 ^oC was successively added Fe power (10 g, 179 mmol). After added, the solution was removed to 80 ^oC, and stirring for 3h. The crude solution was evaporated to dryness and neutralized by addition of $NaHCO_3$ solution (100 mL). The water phase was extracted with ethyl acetate. The combined organic layer was dried (MgSO4), and after evaporation of the volatiles, the resulting compound was used directly for the next step. Triethoxy methane (42 mL, 250 mmol) and $MgSO_4$ (3 g, 25 mmol) was added to the solution of 10b in 100 mL of DMF at 120 ^oC for overnight. The crude solution was evaporated to dryness, and was partitioned between EA and water. The solvent was extracted with ethyl acetate, and the organic layers washed once with water and brine and dried over MgSO4. After evaporation of the volatiles, the resulting compound purification by silica gel column chromatography to obtain 4.23 g (69%) of compound **e** as a brown solid. ^1H NMR (400 MHz, Chloroform-*d*) δ 8.98 (s, 1H), 8.03 (s, 1H), 7.42 – 7.28 (m, 5H), 5.41 (s, 2H).

4-(9-benzyl-9H-purin-2-yl)furan-2-carboxamide (**Roxyl-7**)

Roxyl-7 was prepared by compound **e** with 4-(4,4,5,5-tetramethyl-1,3,2-dioxaborolan-2-yl)furan-2-carboxamide following the procedure of **Roxyl-7**. 67% yeild, white solid. ^1H NMR (400 MHz, DMSO-d_6) δ 9.17 (s, 1H), 8.73 (s, 1H), 8.56 (s, 1H), 8.01 (s, 1H), 7.77 (s, 1H), 7.55 (s, 1H), 7.44 (d, J = 7.5 Hz, 2H), 7.32 (dt, J = 27.7, 7.3 Hz, 3H), 5.52 (s, 2H). ^{13}C NMR (101 MHz, DMSO) δ 159.63, 154.20, 152.06, 149.37, 148.74, 147.42, 146.17, 136.90, 132.61, 129.26, 128.82, 128.46, 128.39, 112.99, 46.75.

Roxyl-8 (CAS: 724737-41-1) was purchased from Specs (Specs, Inc. Zoetermeer, The Netherlands).

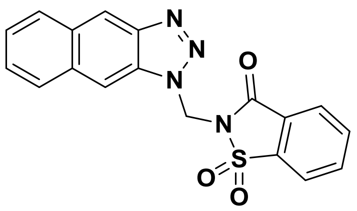

Roxyl-8

Roxyl-9 (CAS: 202582-08-9) was purchased from Specs (Specs, Inc. Zoetermeer, The Netherlands).

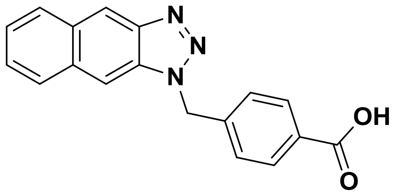

Roxyl-9

Roxyl-10 (cas: 79707-04-3) was prepared by 1,4-naphthoquinone with triazotoluene following the procedure of Su-Ying Wu et al.[1]

^{1}H NMR (400 MHz, DMSO-d_6) δ 8.21 – 8.10 (m, 2H), 8.00 – 7.88 (m, 2H), 7.47 – 7.32 (m, 5H), 6.04 (s, 2H).

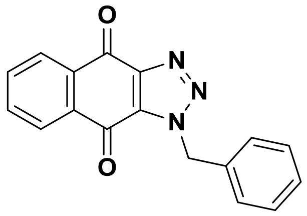

Roxyl-10

Roxyl-11 cas: (667887-37-8) was prepared by 1,4-benzoquinone with triazotoluene following the procedure of Pascal Nebois et al.[2]

^{1}H NMR (400 MHz, DMSO-d_6) δ 7.46 – 7.29 (m, 10H), 6.01 (s, 4H). ^{13}C NMR (101 MHz, DMSO-d_6) δ 168.21, 145.81, 135.12, 134.51, 128.76, 128.45, 128.00, 52.81. HPLC purity 99%.

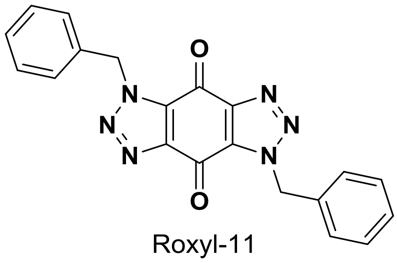

Roxyl-11

13C spectra of compounds Roxyl-11(Roxyl-WL)

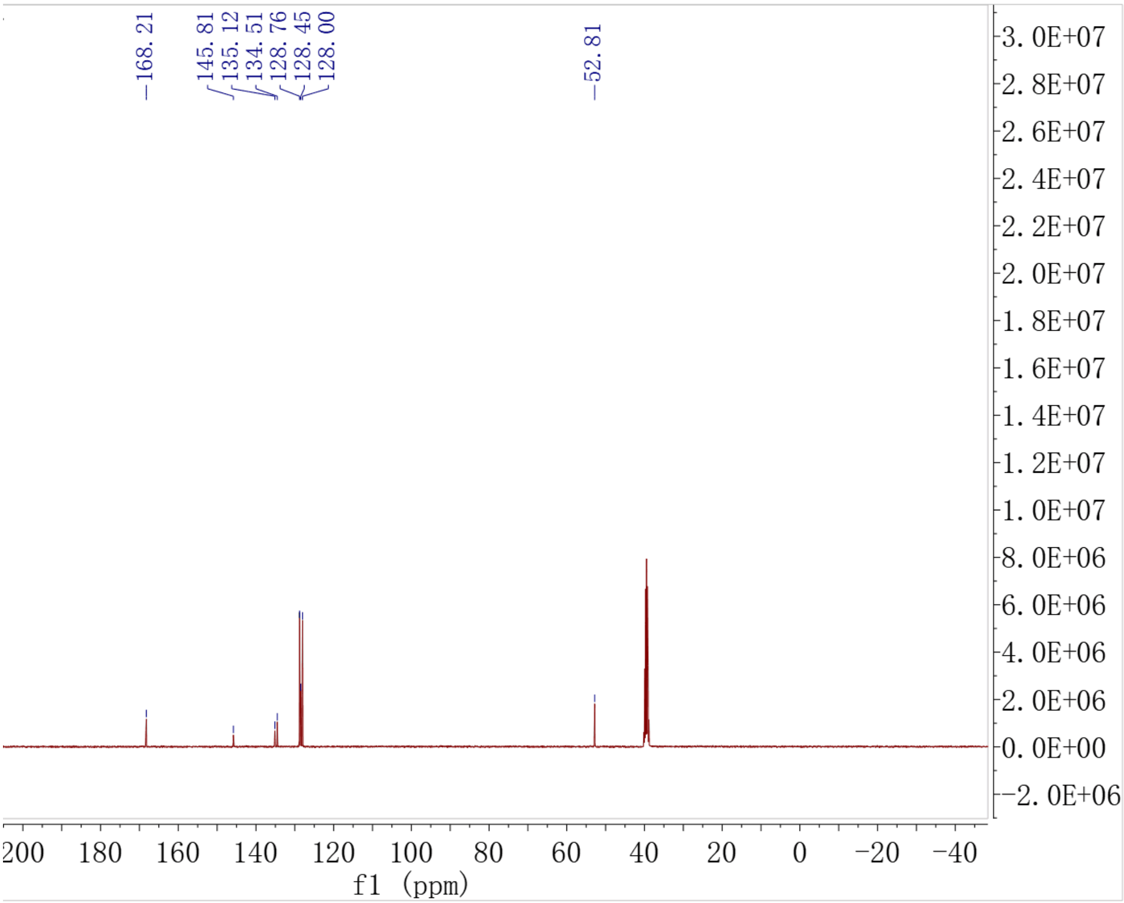

HPLC Purity Analysis for Compound Roxyl-11(Roxyl-WL)

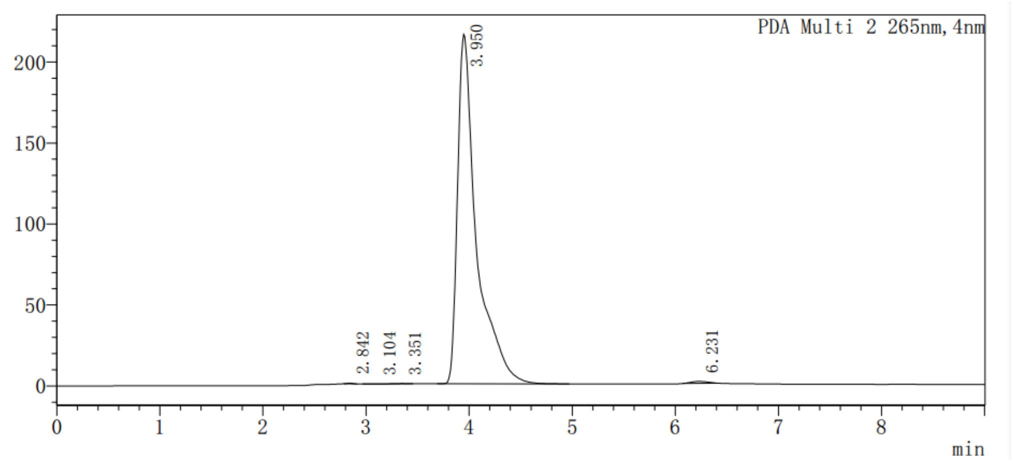

	RT(min)	Area	Area%	Height	Height%
1	2.842	1492	0.054	392	0.180
2	3.104	289	0.010	32	0.015
3	3.351	1029	0.037	122	0.056
4	3.950	2759487	99.383	215634	99.181
5	6.231	14311	0.515	1234	0.568

Roxyl-12 was prepared by 1,4-benzoquinone with methyl 3-(azidomethyl)benzoate following the procedure of **Roxyl-11**. ^{1}H NMR (400 MHz, DMSO-d_6) δ 8.04 (d, J = 1.9 Hz, 1H), 7.93 (dt, J = 7.7, 1.5 Hz, 1H), 7.65 (d, J = 7.7 Hz, 1H), 7.54 (t, J = 7.7 Hz, 1H), 6.09 (s, 2H), 3.85 (s, 3H). ^{13}C NMR (101 MHz, DMSO) δ 168.12, 165.74, 145.76, 135.19, 135.12, 133.06, 130.07, 129.33, 129.20, 128.95, 52.41, 52.28.

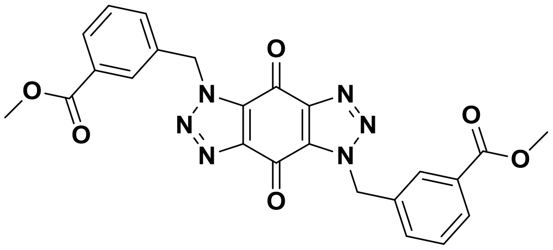

Roxyl-12

Roxyl-13 (CAS: 722461-11-2) was purchased from Specs (Specs, Inc. Zoetermeer, The Netherlands).

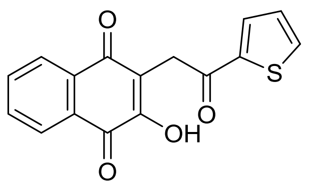

Roxyl-13

Roxyl-14 (CAS: 825611-06-1) was purchased from Specs (Specs, Inc. Zoetermeer, The Netherlands).

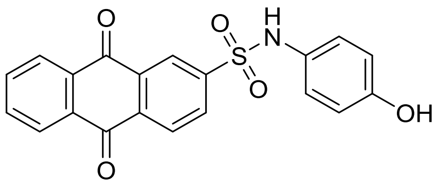

Roxyl-14

Roxyl-15 (CAS: 123475-75-2) was purchased from Specs (Specs, Inc. Zoetermeer, The Netherlands).

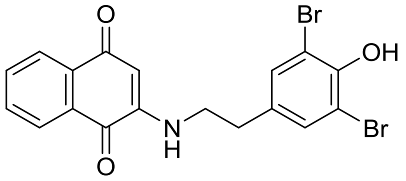

Roxyl-15

Roxyl-16 (CAS: 442553-69-7) was purchased from Specs (Specs, Inc. Zoetermeer, The Netherlands).

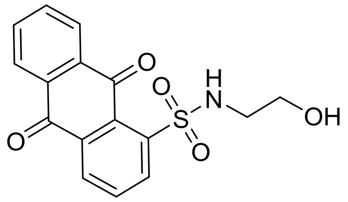

Roxyl-16

Roxyl-17 (CAS: 335205-67-9) was purchased from Specs (Specs, Inc. Zoetermeer, The Netherlands).

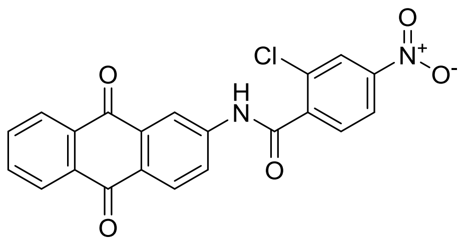

Roxyl-17

Roxyl-18 (CAS: 889940-56-1) was purchased from Specs (Specs, Inc. Zoetermeer, The Netherlands).

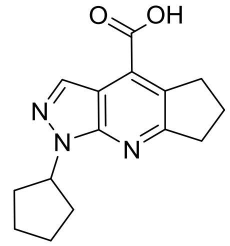

Roxyl-18

Reference:

1. Wu JS, Lin SY, Liao FY, Hsiao WC, Lee LC, Peng YH, et al. Identification of Substituted Naphthotriazolediones as Novel Tryptophan 2,3-Dioxygenase (TDO) Inhibitors through Structure-Based Virtual Screening. J Med Chem 2015, 58(19): 7807-7819.

2. Marminon C, Gentili J, Barret R, Nebois P. Synthesis of N-benzylated indole-, indazole- and benzotriazole-4,7-diones. Tetrahedron 2007, 63(3): 735-739.
